# Family presence policy and waiting room conditions in Japanese intensive care units: A multicentre questionnaire survey

**DOI:** 10.1186/s13054-025-05533-1

**Published:** 2025-07-04

**Authors:** Kasumi Shirasaki, Kaori Shimazu, Shinsuke Hashiuchi, Toru Hifumi, Masaki Okajima, Norio Otani

**Affiliations:** 1 Department of Emergency and Critical Care Medicine, St. Luke’s International Hospital, 9-1 Akashicho, Chuo-ku, Tokyo, 104-8560 Japan; 2 Department of Emergency and Disaster Medicine, Kanazawa University Hospital, 13-1 Takara-machi, Kanazawa, 920-8640 Japan; 3 Department of Nursing, St. Luke’s International Hospital, 9-1 Akashicho, Chuo- ku, Tokyo, 104-8560 Japan; 4 Department of Emergency and General Medicine, Kyorin University School of Medicine, 6-20-2 Shinkawa Mitaka, Tokyo, 181-8611 Japan

**Keywords:** Critical care, Intensive care unit, Post-intensive care syndrome, Questionnaire, Family, Waiting room

## Abstract

**Background:**

After the strict visitation restrictions during the COVID-19 pandemic, the value of family presence has been re-emphasized as an essential part of patient- and family-centred care in the intensive care unit (ICU). The aim of this study was to investigate the status of family presence policies and waiting room conditions in Japanese ICUs.

**Methods:**

A cross-sectional survey of 292 hospitals certified as tertiary medical centres in Japan was performed using a combination of postal and web-based questionnaires. This included 12 questions about institutional characteristics, family presence policies, and waiting room facilities.

**Results:**

Of the 292 tertiary medical centres contacted, 151 (51.7%) responded. Of these, 120 institutions (79.5%) restricted family visiting hours, typically limited to several hours in the afternoon and limited the number of family members at the same time. These restrictions were often relaxed in cases of sudden clinical deterioration or near the end of the patient’s life. In addition, 144 institutions (95.4%) had family waiting rooms for ICUs, and most provided Table (76.4%) and chairs (96.5%). However, only a few offered amenities such as books and magazines (13.9%), napping areas (10.4%), cooking facilities (3.5%), shower rooms (2.1%), or refrigerators (0.7%). Moreover, only 47 institutions (32.6%) ensured adequate privacy in their waiting rooms. There were no significant differences in the family presence policies or waiting room conditions depending on the number of ICU beds, except for the location of the family waiting room.

**Conclusions:**

Most ICUs in Japan restricted family visiting hours to several hours in the afternoon and limited the number of family members at the same time. Moreover, family waiting rooms often lack essential amenities and do not sufficiently protect privacy.

**Supplementary Information:**

The online version contains supplementary material available at 10.1186/s13054-025-05533-1.

## Background

The presence of family members in the intensive care unit (ICU) is increasingly recognized as essential for improving patient outcomes, including a reduced risk of delirium and better continuity of care after ICU discharge [1, 2]. Considering these benefits, ICU family presence policies should primarily be designed to support the needs of patients. However, family members often experience psychological symptoms such as anxiety, depression, and posttraumatic stress disorder (PTSD), collectively referred to as post intensive care syndrome-family (PICS-F) while their loved one is in the ICU [3, 4]. Therefore, healthcare professionals must also attend to the basic needs and comfort of family members. ICU family presence policies and family waiting room conditions are important components of family-centred care, and the importance of structured support to family caregivers has been emphasized in recent years [2, 5–8].

The Institute for Patient- and Family-Centered Care (IPFCC) and Planetree International advocates refer to close family members not as “visitors” but as “essential care partners,” and they recommend promoting more flexible family presence at the bedside [9, 10]. A study of two ICUs in Australia reported that family satisfaction was impacted by communication with medical staff, waiting room facilities, and visiting times [11]. Furthermore, in Australia and New Zealand, nationwide surveys of ICU family presence policies and family waiting room conditions identified threats to implementing family-centred engagement and care even prior to the COVID-19 pandemic [12]. These pre-existing challenges gained renewed attention globally because strict limitations on family presence, though necessary for infection control, had a psychological impact on patients, families, and ICU team members in the COVID-19 era [13–15]. However, no studies have investigated current ICU family presence policies or family waiting room environments in Japan.

The aim of this study was to investigate the status of family presence policies and waiting room conditions in Japanese ICUs.

## Methods

This was a cross-sectional survey to investigate the practices of family presence policies and family waiting room conditions in Japanese ICUs. The questionnaire was mixed multiple-choice and short-response answers, completed via post or an online link (using a QR code). The present study is reported according to the Consensus-Based Checklist for Reporting of Survey Studies (CROSS) guideline for surveys [16]. The web-based survey was created and managed using the Research Electronic Data Capture (REDCap), a secure, web-based software platform hosted by St. Luke’s International University. REDCap is widely used in academic research for electronic data capture (EDC) and includes robust security features to prevent unauthorized access, and only authorized members of the research team had access to the data. The study adhered to the ethical guidelines set forth in the Declaration of Helsinki and received approval from the Institutional Review Board of St. Luke’s International Hospital (Approval No. 23-R024).

### **Participants and recruitment**

The present study targeted ICUs at hospitals certified as tertiary medical centres in Japan. Japanese hospitals are classified into three levels of medical institutions according to the severity of patients transported by emergency services. A “primary” emergency centre deals with patients who can be managed as outpatients, a “secondary” emergency centre deals with patients who can be managed as inpatients on a general medical floor, and a “tertiary” medical centre deals with patients who need to be managed in the operating room or the ICU. Paramedics triage patients at the scene and transport them to the appropriate facility level. Consequently, patients admitted to ICUs in tertiary medical centres tend to be more severely ill. Hospital addresses were obtained from the list of all tertiary medical centres published on the Ministry of Health, Labour and Welfare website.

In August 2024, a self-addressed questionnaire was mailed to the director of the Emergency and Critical Care Centre at each institution requesting that the questionnaire be completed and returned by 31 September 2024. ICU medical directors of each institution were asked to complete the questionnaire or delegate it to an appropriate person. The respondent could be a physician, nurse, or administrative staff member, provided that they were familiar with the details of family presence policies and family waiting rooms. Only one response was collected per centre. If the institution had multiple ICUs or family waiting rooms, they were asked to provide a general description of a typical ICU or waiting room. All respondents could select whether to return their responses by post or respond online. Initial consent was indicated through a checkbox on the survey form, which participants marked to signify their willingness to participate in the study.

### Questionnaire

The questionnaire was developed by the research team, which included intensive care clinicians and nurses based on the questionnaire used in the survey by Tabah et al. [12], with modifications to reflect the Japanese clinical context. Although no formal pilot testing was conducted, the draft questionnaire was reviewed by the research team. They completed it and provided feedback to ensure that the intent of each item was clear and that no questions were ambiguous. The questionnaire included a total of 12 questions: two concerned the characteristics of the institutions (total number of beds and number of ICU beds); eight were related to the family waiting room and available services (including facilities to sleep on site); and two addressed family presence (including visiting hours and the possibility to extend them, and the maximum number of family members allowed at one time). Key terms were defined within the survey: “family waiting room” was defined as a place where family members of patients admitted to the ICU could spend time, for example, when waiting for a physician to explain the patient’s condition; and “private waiting room” was defined as an area where families could wait without the risk of being seen or overheard by others. The estimated completion time was 5 to 10 min.

### Data collection

Responses submitted online were recorded directly into the REDCap system. The data from questionnaires returned via post were entered into the REDCap system by one researcher and verified by a second researcher to minimize human error in data entry. To prevent multiple responses in the web-based survey, respondents were required to enter the name of their institution. For paper-based surveys, the return envelopes were pre-labelled with the institution name in the sender’s field to ensure that the institution could be identified even if it was not written on the form. The research team cross-checked entries in the REDCap system to ensure that no more than one response was received from each institution. When exporting the data from REDCap for analysis, all institution names were removed to ensure the anonymity of the participants. The questionnaire text can be accessed in the supplemental materials (Supplemental file 1).

### Statistical analysis

For continuous variables, results are presented as medians with interquartile ranges, and categorical variables are presented as proportions. A univariate analysis was conducted to compare the differences in family presence policies and family waiting room conditions between institutions stratified by total hospital bed count (≥ 500 beds vs. < 500 beds) and by number of ICU beds (≥ 10 beds vs. < 10 beds). A two-sided *p* value of less than 0.05 was considered significant in the analyses. Statistical analyses were performed using JMP Version 18 statistical software (SAS Institute, Cary, NC). Missing data were excluded from the analyses.

## Results

Of the 292 tertiary medical centres that were initially contacted, 151 (51.7%) returned the questionnaire and provided consent for the use of the survey responses.

### Characteristics of institutions

**Fig. 1:**
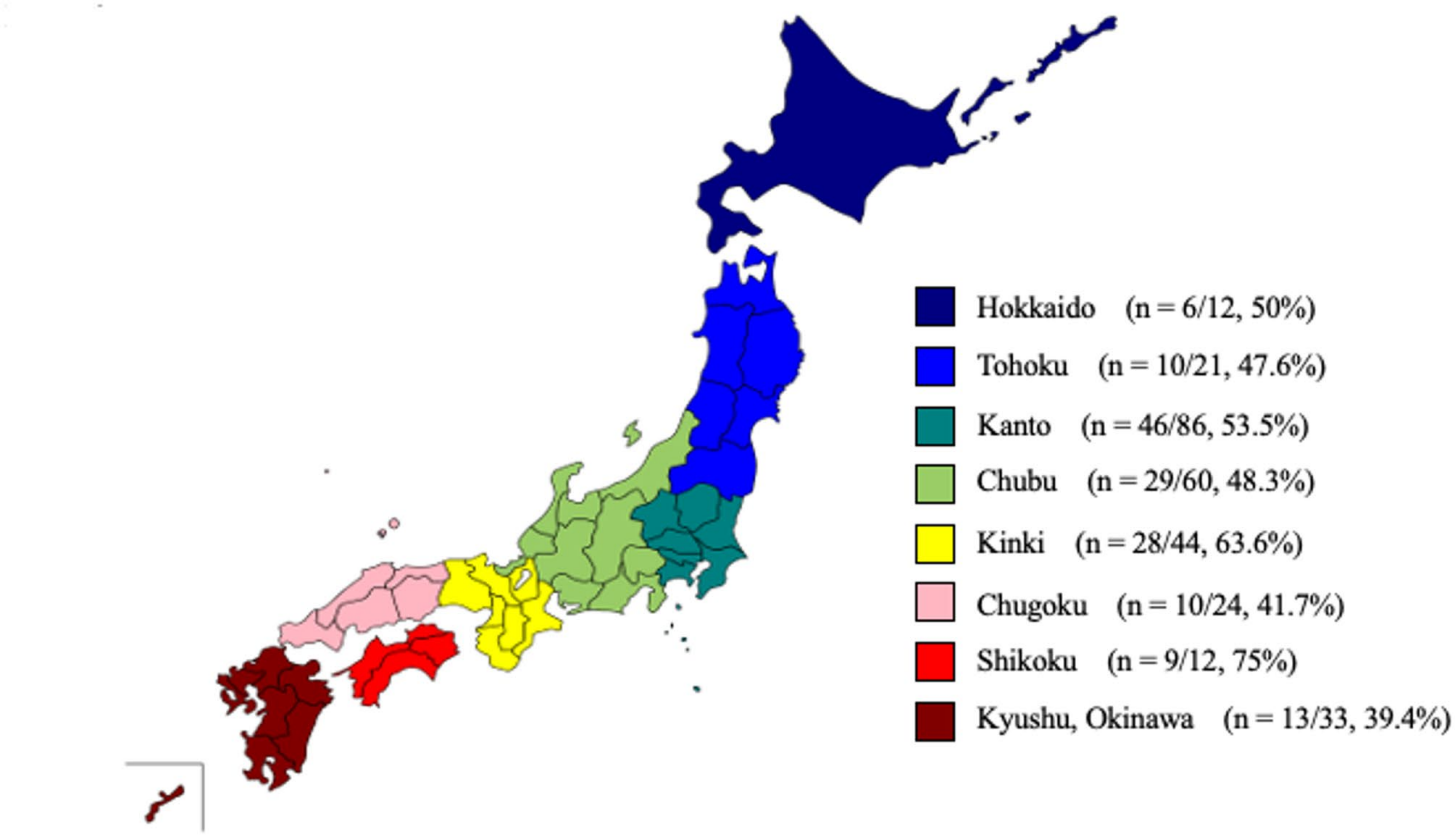
Geographic distribution of participating institutions and regional response rates. Each region is color-coded, and the number of responding institutions relative to the total number of tertiary medical centres is indicated

The characteristics of the institutions that responded to the questionnaire are shown in Table 1. The majority of hospitals were community-based (75.2%) and had ≥ 10 beds (72.8%). The institutions were distributed across Japan. The geographic distribution of participating institutions and the regional response rates are shown in Fig. 1.

**Table 1:** Baseline characteristics of institutions

Characteristics	Institutions (*n* = 151)
**Hospital classification**	
University Hospital, n. (%)	37 (24.8%)
Community Hospital, n. (%)	112 (75.2%)
**Hospital beds**	
< 500, n. (%)	52 (34.4%)
≥ 500, n. (%)	99 (65.6%)
**ICU beds**	
< 10, n. (%)	41 (27.2%)
≥ 10, n. (%)	110 (72.8%)
**Family waiting rooms**	
No family waiting room, n. (%)	7 (4.6%)

Data are presented as numbers and percentages for categorical variables

ICU, intensive care unit

### Family presence policy

**Fig. 2:**
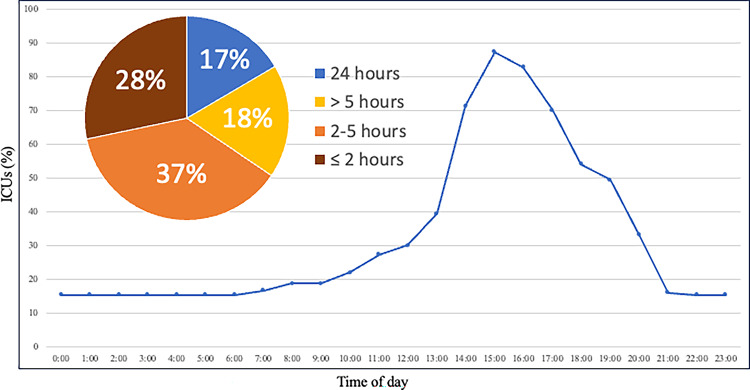
Relative proportions of amenities and equipment available in family waiting rooms

**Fig. 3:**
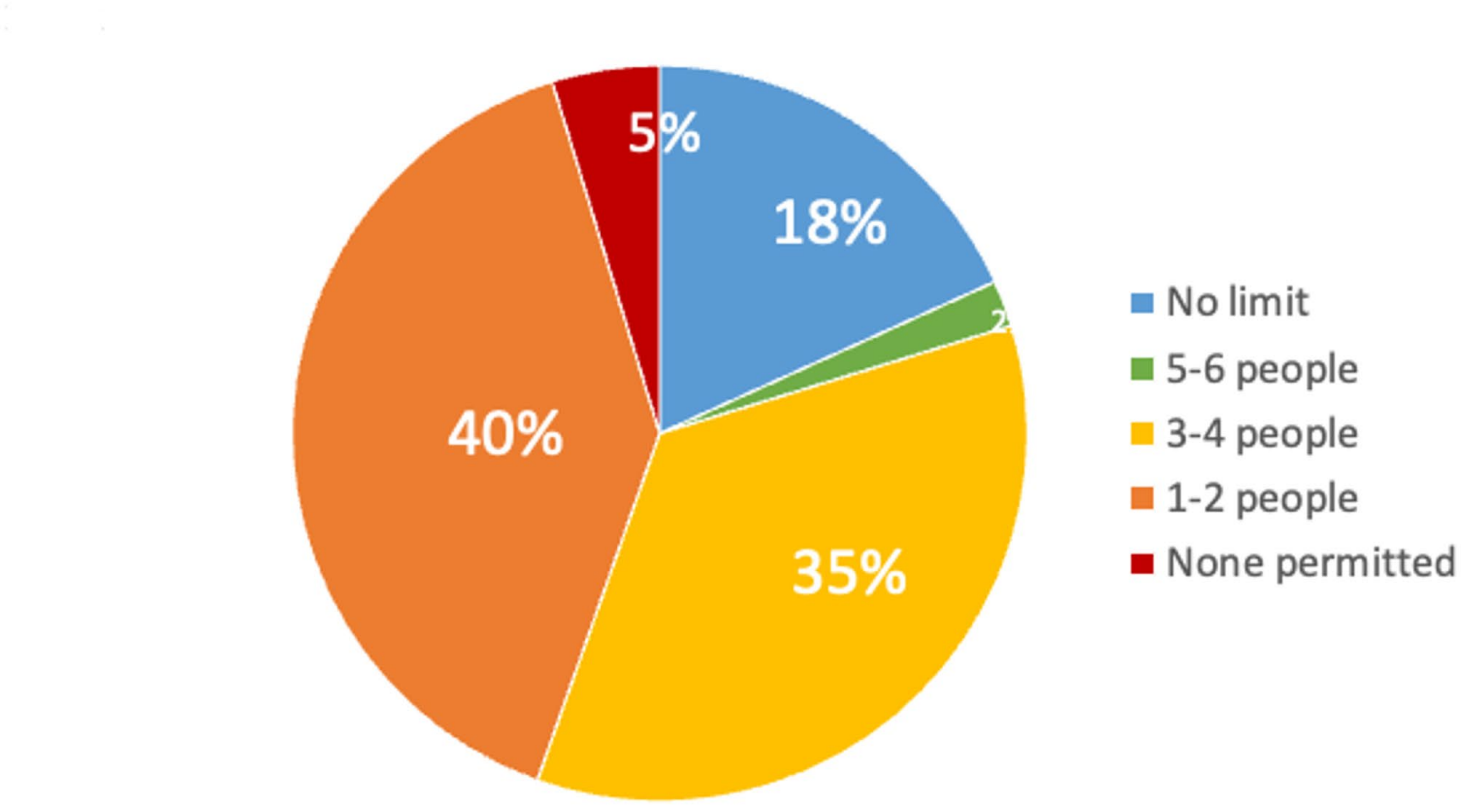
Visitation hours permitted for family members of ICU patients. The line-by-line graph shows the percentage of ICUs allowing family visits, with peak visiting times in the afternoon. Each data point represents the proportion of ICUs open to visitors per hour

The specific details of family presence policy at each institution are summarised in Table 2. Twenty-four institutions (15.9%) permitted 24-hour family presence. In contrast, 120 institutions (79.5%) imposed restrictions on visiting hours, and visiting hours were limited to a few hours, primarily allowing visits in the afternoon (Fig. 2). Seven institutions (4.6%) prohibited family presence entirely. On the other hand, several institutions indicated that they would relax family presence restrictions under certain circumstances, especially near the end of the patient’s life (79.5%), in cases of clinical deterioration (80.8%), or at the request of a family member (32.5%). However, only approximately half of the institutions indicated that they provided a place for family members to rest overnight if they were called to the hospital at night. Most institutions (81.3%) limited the number of visitors in the ICU at the same time (Fig. 3). No significant differences were observed depending on the number of hospital beds and ICU beds.

**Table 2:** Details of family presence policy

Characteristics	All institutions (*n* = 151)	Institutions with ≥ 10 ICU beds (*n* = 108)	Institutions with < 10 ICU beds (*n* = 36)	*p*-value
**Time allowed for families to visit ICU patients**				
24-hour visitation permitted, n. (%)	24 (15.9%)	17 (15.5%)	7 (17.1%)	0.07
Restricted visitation hours, n. (%)	120 (79.5%)	88 (80.0%)	32 (78.1%)	
Visitation prohibition, n. (%)	7 (4.6%)	5 (4.6%)	2 (4.9%)	
**Visiting hours**				
Visiting hours permitted (minutes), median. [IQR]	180 [120–300]	180 [120–300]	180 [120–300]	0.72
**Situations in which family presence policy may be extended**				
Near end of life, n. (%)	120 (79.5%)	88 (80.0%)	32 (78.1%)	0.82
Clinical deterioration, n. (%)	122 (80.8%)	88 (80.0%)	34 (82.9%)	0.82
On family request, n. (%)	49 (32.5%)	38 (34.6%)	11 (26.8%)	0.44
**Places for overnight stays if a family member is called in at night**				
Cots at the patient’s bedside, n. (%)	23 (15.2%)	19 (17.3%)	4 (9.8%)	0.32
Dedicated rooms in the hospital, n. (%)	31 (20.5%)	23 (20.9%)	8 (19.5%)	1.00
Accommodations provided by the hospital, n. (%)	6 (4.0%)	4 (3.6%)	2 (4.9%)	0.67
No place to sleep, n. (%)	77 (51.0%)	56 (50.9%)	21 (51.2%)	1.00
Other, n. (%)	21 (13.9%)	15 (13.6%)	6 (14.6%)	1.00
**Restrictions on the number of family members at one time**				
Limit on the number of family members to the ICU, n. (%)	122 (81.3%)	89 (80.9%)	33 (82.5%)	1.00
Maximum number of family members at one time, median. [IQR]	2 [2–3]	2.5 [2–3]	2 [2–3]	0.67

Data are presented as numbers and percentages for categorical variables, otherwise as median and interquartile range

ICU, intensive care unit; IQR, interquartile range

### Family waiting room conditions

**Fig. 4:**
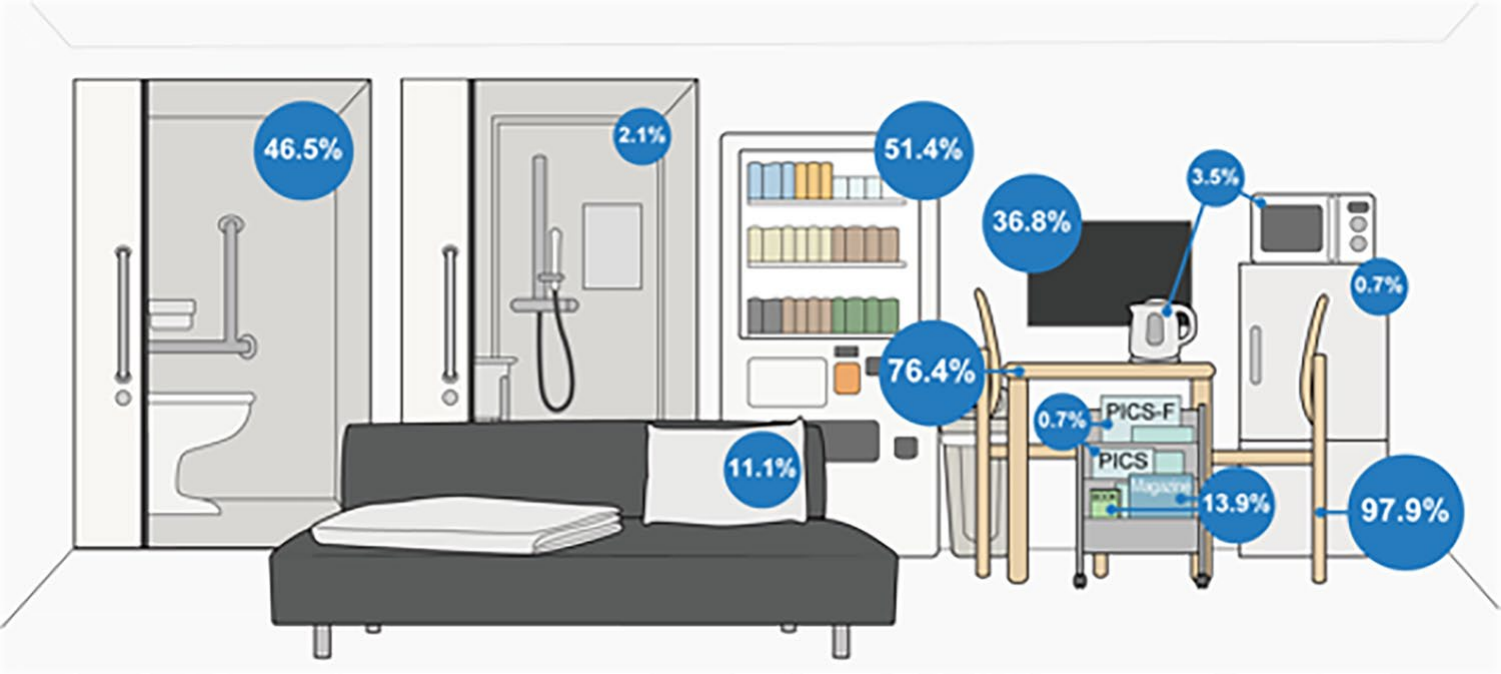
Number of family members permitted to visit ICU patients simultaneously

The specific details of the 144 institutions that reported having a family waiting room are given in Table 3. Over half of the family waiting rooms (59.9%) were smaller than 50 m^2^. There were no significant differences in the sizes of the waiting rooms, even between institutions with more or less than 10 ICU beds (*p* = 0.15). The proportion of units providing each waiting room amenity is presented in Fig. 4. Except for the presence of a toilet room, which was more common in ICUs with ≥ 10 beds (50.9% versus 30.6% respectively, *p* = 0.04), there were no differences found in availability of amenities by size of unit. Regarding privacy, 47 institutions (32.6%) reported having a private room or partitions to prevent interactions with other patients’ families. There were no differences (all *p* values > 0.05) in any variables between hospitals with more or less than 500 beds.

**Table 3:** Characteristics of family waiting rooms

Characteristics	All institutions (*n* = 144)	Institutions with ≥ 10 ICU beds (*n* = 108)	Institutions with < 10 ICU beds (*n* = 36)	*p*-value
**Location of the family waiting room**				
Part of the ICU/Adjacent to the ICU, n. (%)	94 (65.0%)	75 (69.4%)	19 (52.8%)	0.10
Away from the ICU, but on the same floor, n. (%)	48 (33.0%)	34 (31.5%)	14 (38.9%)	0.42
On a different floor from the ICU, n. (%)	4 (2.8%)	1 (0.9%)	3 (8.3%)	0.04
**Privacy in the family waiting room**				
Completely private rooms or partitions to avoid encountering other patients’ families, n. (%)	47 (32.6%)	35 (32.4%)	12 (33.3%)	1.00
Private rooms but shared with other patients’ families, n. (%)	47 (32.6%)	34 (31.5%)	13 (36.1%)	0.68
Open space, like a lobby, visible to others, n. (%)	55 (38.2%)	42 (38.9%)	13 (36.1%)	0.84

Data are presented as numbers and percentages for categorical variables

ICU, intensive care unit

## Discussion

The present study investigated the status of family presence policies and waiting room conditions in Japanese ICUs. Most institutions restricted family visiting hours to several hours in the afternoon and limited the number of family members permitted simultaneously. However, it was noted that many institutions relaxed family presence restrictions in specific cases, such as sudden deterioration in the patient’s condition or near the end of life. In contrast, only approximately half of the institutions indicated that they provided a place for family members to rest overnight if they were called to the hospital at night. In addition, most ICU family waiting rooms were furnished with basic furniture such as tables and chairs, whereas amenities such as microwaves, other cooking equipment, or areas for napping were less common. Furthermore, fewer than half of the institutions provided waiting rooms that adequately protected the privacy of families. There were no significant differences in the family presence policies or waiting room conditions depending on the number of ICU beds, except for the location of the family waiting room.

Despite broad consensus that liberalization of the family presence policy in the ICU improves the care and experience of patients and families [2], ICU family presence policies differ across regions: whereas ICUs in North America and Oceania have moved toward more liberal, open visitation, institutions in Europe commonly enforce limitations on visiting hours and the number of family members permitted simultaneously [17–19]. Based on the present study, family presence policies in Japan were more like European practices than those in North America and Oceania. In North America and Oceania, family members are often considered essential care partners and actively included in ICU care [20]. This perspective has been supported at a national level, particularly through initiatives such as the “Better Together” campaign [21]. In contrast, such a concept has not been widely embraced in Japanese ICUs, where families are rarely involved in clinical rounds or direct patient care. Even before the COVID-19 pandemic, many Japanese ICUs had restricted family presence, and 75% of ICUs have a time limit for a single visitation [22]. This cultural difference may explain why strict family presence policies have continued in Japan. In addition, they are often asked to wait outside the ICU during routine care. As a result, it is likely that most ICUs in Japan have established designated family waiting rooms.

However, the results of the present study indicate that Japanese ICUs face challenges related to the provision of privacy in family waiting rooms and the availability of amenities that support a comfortable stay. The guidelines from the United States on ICU design recommend that family waiting rooms be located near the ICU and structured to ensure privacy [8, 23]. In addition, it is recommended that vending machines, microwaves, refrigerators, and coffee makers be provided when hospital coffee shops or food services may not be available [8, 23]. Most ICUs in the Netherlands have facilities for the family members that include catering facilities such as coffee, tea, and meals [24]. Although similar recommendations are also found in Japanese guidelines [25], the present findings suggest that these standards are not consistently implemented across Japanese ICUs. Japan has a relatively low proportion of ICU beds and a limited number of trained intensivists compared with other developed countries [26]. According to a previous report, the low prevalence of ICU services in Japan may be attributed to a weak historical tradition, insufficient public and social awareness, and limited governmental funding [26]. As a result, it is possible that the comfort of family waiting rooms has not been a major focus until recent years in Japan.

This is the first report to assess the issue of family presence policies and waiting room conditions in Japan. The two aspects investigated, family presence policies and the conditions of family waiting rooms, are closely linked to family satisfaction [11]. Descriptive studies of family satisfaction have reported that families are often dissatisfied with the environment of ICU waiting rooms, such as privacy and amenities [11, 27]. Since family satisfaction has been identified as a potential risk factor for PICS-F, improving satisfaction may contribute to the prevention of PICS-F. For example, essential care partners could be at the bedside for 24 h, not only in life-threatening situations, whereas visitation by others who are not involved in care could remain limited. In terms of privacy, even if a waiting room is situated in an open area such as a lobby, installing partitions could easily increase privacy, preventing families from being in direct view of others [8]. Addressing these issues is likely to be an important part of future interventions aimed at preventing PICS-F in Japan.

The present study has several limitations. First, the response rate for the questionnaire was 51.7%, and the voluntary nature of the survey may have introduced sampling bias. Second, the reliance on self-administered questionnaires means that there is a possibility that the data obtained may not be accurate. Third, the present study targeted ICUs at hospitals certified as tertiary medical centres in Japan, and it may not be possible to generalise the results, because ICUs in hospitals certified as primary or secondary emergency centres were not included. However, since patients admitted to ICUs in tertiary medical centres tend to be more severely ill, the hospitals targeted represent institutions with a potentially higher need for family-centred care. Finally, whether family presence policies had changed before and after the COVID-19 pandemic was not specifically investigated. However, previous reports on ICU family presence policies in Japan before the COVID-19 pandemic showed similar restrictions, suggesting that the policies observed in the present study were not significantly influenced by the pandemic.

## Conclusions

Most ICUs in Japan restricted family visiting hours to several hours in the afternoon and limited the number of family members at the same time. Moreover, family waiting rooms often lack essential amenities and do not sufficiently protect privacy.

## Supplementary Information

Supplementary Material 1.

## Data Availability

No datasets were generated or analysed during the current study.
