# Supplementary material for: Family presence policy and waiting room conditions in Japanese intensive care units: A multicentre questionnaire survey
Source: Crit Care. 2025 Jul 4;29:278. doi: 10.1186/s13054-025-05533-1 (PMC12228183; doi:10.1186/s13054-025-05533-1)
Supplement: Supplementary file 1 — Supplementary Material 1. [file 13054_2025_5533_MOESM1_ESM.docx]

**Supplemental Table 1. Questionnaire text**

1. **What is the bed capacity at your institution?**

□ Less than 300 bed

□ 300 to less than 500 beds

□ 500 to less than 1000 beds

□ 1000 beds or more

1. **What is the bed capacity of the ICU at your institution? If your institution has multiple ICUs, please provide the total number of ICU beds.**

- Less than 6 beds
- 6 to less than 10 beds
- 10 to less than 20 beds
- 20 beds or more

1. **Does your institution have a family waiting room for ICU patients' families? (This refers to a** **place where family members of patients admitted to the ICU can spend time, for example, when waiting for a physician to explain patient’s condition. It does not matter whether it is a private room or not).**

- Yes
- No

1. **If there is a family waiting room, where is it located?**

- Part of the ICU/ Adjacent to the ICU
- Away from the ICU, but on the same floor
- On a different floor from the ICU

1. **What is the size of the family waiting room?**

□ < 50 m^2^

□ 50 to 100 m^2^

□ 101 to 150 m^2^

□ ≥ 151 m^2^

1. **Does the family waiting room have the following facilities? (This includes facilities within a 1–2 minute walking distance from the waiting room).**

- Table □ Chair □ Vending machine □ Cooking facilities such as a microwave and a kettle
- Refrigerator　□ Television　□ Books and magazines　□ Restroom　□ Shower room
- Napping area (including cots)　 □ Information booklet about PICS or PICS-F

1. **What is the level of privacy in the family waiting room?**

- Completely private rooms or partitions to avoid encountering other patients’ families
- Private rooms but shared with other patients’ families
- Open space, like a lobby, visible to others

1. **Is the ICU open for 24-hour visitation?**

- Yes
- No

**8-2. If no, please specify the visiting hours (e.g., from 08:00 to 10:00).**

　From __________to__________

**8-3. Under what circumstances are visiting hours extended?**

□ Near end of life

□ Clinical deterioration

□ On family request

1. **If families are called to the hospital during the night (e.g., upon admission or in case of sudden clinical deterioration), is there a place in the hospital where they can sleep?**

□ Cots at the patient's bedside

□ Dedicated rooms in the hospital

□ Accommodations provided by the hospital

□ No place to sleep

□ Other

1. **Is there a limit on the maximum number of visitors allowed per patient at a time?**

- Yes, maximum number: _________________
- No

1. **Is there a dedicated room or area for doctors to explain the patient’s condition to the family?**

- Yes
- No

1. **Please share any special considerations or improvements that your institution has implemented regarding family waiting rooms.**

_____________________________________________________________________________________
